# Coronary Artery bypass grafting and/or valvular surgery in patients with previous pneumonectomy

**DOI:** 10.1186/1749-8090-7-110

**Published:** 2012-10-10

**Authors:** Alexander Fragkidis, Alexander Dimitriou, Dimitrios Dougenis

**Affiliations:** 1 Department of Cardiothoracic Surgery, Patras University School of Medicine, Rion, 26500, Greece

## Abstract

There is a lack of data regarding heart surgery on patients who have been previously pneumectomized. These patients pose unique challenges and surgical management may necessitate deviations from standard methods in the perioperative course. To summarize the available knowledge and to assess the optimal methods, we reviewed all reported patients with prior pneumonectomy who were subjected to coronary artery bypass grafting and/or valve surgery.

In a Medline search from 1966 to May 2011 carefully undertaken, we identified 22 articles, including 29 patients who underwent 30 operations: CABG 70%, valvular surgery 23%, and combination 7%. Severe morbidity was 37% and 30-day mortality 13%.

Although postoperative morbidity and mortality remain higher in previously pneumectomized patients undergoing coronary artery bypass grafting and valvular surgery, the gathered experience up to date suggests that a carefully planned surgical strategy, along with the use of advanced modern techniques may reduce morbidity and improve final outcome.

## Background

Previous pneumonectomy is a rare comorbidity among patients undergoing open heart surgery for ischemic or valvular heart disease and poses unique intraoperative and postoperative challenges. Although several authors have proposed experience-based standards and considerations concerning surgical strategies for these patients, official guidelines have yet to be established. In this study, our aim was to review the current data and established suggestions and general principles in cardiac surgery on the pneumectomized patients.

## Methods

We conducted a MEDLINE search from 1966 to May 2011 for available literature on patients with previous pneumonectomy who underwent open heart surgery for coronary artery bypass grafting (CABG) and/or valve repair or replacement, alone or in combination. The collected data was evaluated in detail and the site of operation, the type of cardiac procedure as well as the relative demographic data and the final outcome for each patient were individually presented.

## Results

The results are summarized in Table 1. We identified 29 patients whose cases were reported in 22 articles [1-22]. The mean age of the patients was 67 years, ranging from 48 to 83 years; 72% of patients (n = 21) were male; 66% of patients (n = 19) had undergone left pneumonectomy; mean elapsed time between pneumonectomy and operation was 19 years, ranging from 9 months to 51 years.

**Table 1:** Summary of 29 Reported Patients In The Literature With Previous Pneumonectomy who Underwent CABG and/or Valve Replacement/Repair Surgery*

**Author/Year**	**Patient No.**	**Age/Gender/ Site/Elapsed time**	**Preoperative Data**	**Operation**	**Postoperative Complications/Course**
Lecharpentier et al [1]/ 1988	1	66/M/Left/12 y	**D**LCO, (78%); PaO_2_, 80 mmHg; PaCO_2_, 43 mmHg; pH, 7.35	CABG: LIMA → LADA (median sternotomy, on-pump)	Inotropic support, respiratory failure, pneumothorax/ Discharged on day 10
	2	66/F/Right/36 y	**D**LCO, (95%)	Mitral valve replacement, tricuspid valve annuloplasty (median sternotomy, on-pump)	Inotropic support, atrial fibrillation/ Died on day 12
Medalion et al [2]/1994	3	70/F/Left/40 y	FEV_1_, 0.95 L (45%); FVC, 1.3 L (52%)	CABG: 3 SVGs, LIMA (median sternotomy, on-pump)	None/Discharged on day 11
Shibata et al [3]/1994	4	67/M/Left/13 y	FEV_1_, (77%); FVC, (55%); room air PaO_2_, 92 mmHg; PaCO_2_, 45 mmHg	CABG: 3 SVGs (median sternotomy, on-pump)	None /Discharged on day 57
Berrizbeitia et al [4]/1995	5	61/M/Right/42 y	FEV_1_, 0.59 L (21%); FVC, 1.27 L (32%); **D**LCO, (48%); room air PaO_2_, 77 mmHg; PaCO_2_, 48 mmHg; pH, 7.40; TLC, 2.97 (49%); PAP, 25/14 mmHg	CABG: 3 SVGs → LADA, OMB, PDA (median sternotomy, on-pump)	None/Discharged on day 18
Izzat et al [5]/1995	6	65/M/Right/10 y	Not specified	Mitral valve replacement (median sternotomy, on-pump)	Postoperative hypotension;pneumothorax; mediastinitis; sepsis/Died on day 12
Demirtas et al [6]/1995	7	63/M/Left/20 y	FEV_1_, 1.09 L (36%); FVC, 1.36 L (36%); room air PaO_2_, 68 mmHg; PaCO_2_, 36 mmHg; PAP, 28/11 mmHg	CABG: LIMA → LADA SVG → 1OMG (median sternotomy, on-pump)	None /Discharged on day 7
Soltanian et al [7]/1998	8	70/F/Left/19 y	FEV_1_, 1.06 L; FVC, 1.58 L	CABG: SVG → LADA (left thoracotomy, on-pump)	Respiratory failure; pulmonary embolism; pneumonia /Diedon day 6
Lippmann and Au [8]/2000	9	68/M/Left/15 y	FEV_1_, 1.65 L (56%); FVC, 2.30 L (60%)	CABG: 3 SVGs (median sternotomy, on-pump)	Postoperative bleeding requiring re-exploration; respiratory failure; atrial fibrillation; hemothorax/ Discharged on day 48
	10	73/M/Left/22 y	FEV_1_, 1.65 L (53%); FVC, 2.40 L (58%)	CABG:SVG, LIMA (median sternotomy, on-pump)	None / Discharged on day 9
Golbasi et al [9]/2001	11	58/M/Right/9 mo	FEV_1_, 1.42 L (50%); FVC, 1.57 L (44%); room air PaO_2_, 76 mmHg; PaCO_2_, 38 mmHg; pH, 7.51; PAP, 35/18 mmHg	CABG: 3 SVGs → LADA, 1OMB, PDA (median sternotomy, on-pump)	Respiratory failure / Discharged on day 12
Diab et al [10]/2001	12	64/M/Right/6 y	PaO_2_, 68 mmHg; PaCO_2_, 46 mmHg; pH, 7.38	CABG: SVG (median sternotomy, on-pump)	Pneumothorax / Discharged on day 20
El-Hamamsy et al [11]/2003	13	65/F/Right/51 y	FEV_1_,0.86 L (36%); FVC, 1.37 L (44%)	Mitral valve replacement, tricuspid valve annuloplasty (median sternotomy, on-pump)	None / Discharged on day 6
	14	71/F/Right/50 y	FEV_1_, 0.60 L (28%); FVC, 0.75 L (27%)	CABG: SVGs (median sternotomy, off-pump)	None / Discharged on day 7
Kumar et al [12]/2003	15	70/M/ Left/ 15 y	FEV_1_, 1.74 L; FVC, 3.18 L; room air PaO_2_, 62 mmHg; PaCO_2_, 37 mmHg; K_CO_, (72%)	CABG: LIMA → LADA SVG → PDA (median sternotomy, off-pump)	None / Discharged on day 5
Erdil et al [13]/2004	16	51/M/ Right/ 17 y	FEV_1_, 1.5 L (45%); FVC, 1.9 L (43%); **D**LCO, (71%); PaO_2_, 72.6 mmHg; PaCO_2_, 38.7 mmHg; pH, 7.47	CABG: RA → RCA RA(Y) → LADA, OMB (median sternotomy, on-pump)	None / Discharged on day 10
Shanker et al [14]/2005	17	80/M/ Left / 27 y	FEV_1_, 1.0 L (46%); **D**LCO/VA, (53%); mean PAP, 54 mmHg	Mitral valve repair, aortic valve replacement, CABG: SVG → LADA, diagonal branch (median sternotomy, on-pump)	None / Discharged on day8
Bernet et al [15]/ 2006	18	58/M/ Right /3 y	FEV_1_, 1.41 L (59%); FVC, 2.22 L (59%)	CABG: LIMA → LADA SVG → LCX, RCA (median sternotomy, on-pump)	None / Discharged on day 7
MH Us et al [16]/2006	19	74/M/ Left /15 y	FEV_1_, 1.35 L (45%); FEV_1_/FVC, 0.65 (60%), PaO_2_, 64 mmHg; PaCO_2_, 45 mmHg; pH, 7.42	Mitral valve replacement, subaortic membrane resection (median sternotomy, on-pump)	None / Discharged on day 7
MH Us et al [17]/2010	20	65/M/ Left /8 y	FEV_1_, 1.30 L (45%); FEV_1_/FVC, 0.60 (50%); PaO_2_, 60 mmHg; PaCO_2_, 48 mmHg	CABG: SVG → LADA, circumflex (left thoracotomy, off-pump)	None / Discharged on day 5
Stoller et al [18]/2007	21	54/F/ Left /3 y	FEV_1_, 1.63 (61%); FVC, 2.03 (61%)	CABG: 3 SVGs → RCA, LADA, LCX (emergent median sternotomy, on-pump)	Respiratory failure; pneumonia / Discharged on day 26; admitted 8 y later with respiratory and congestive heart failure
	22	48/M/ Left /18 y (before first surgery), 26 y (before second surgery)	First surgery: PAP, 65/30 mmHg; CO, 6.7 L/min. No pulmonary function tests available due to emergency of situation	Tricuspid and mitral valve repair (re-sternotomy, on-pump)	Atrial fibrillation / Discharged on postoperative day 13
			Second surgery: FEV_1_, 1.36 L (37%); FVC, 1.94 (42%); FEV_1_/FVC, 0.71 (85%); RVSP, 52 mmHg		
	23	71/M/ Left /7 y	FEV_1_, 1.17 L (33%); FVC, 1.86 L (40%); FEV_1_/FVC, 0.63 (83%); room air PaO_2_, 79 mmHg; PaCO_2_, 41 mmHg; pH, 7.46	Mitral valve replacement, tricuspid valve annuloplasty (median sternotomy, on-pump)	Hypotension and renal failure; atrial fibrillation / Survived (no further data)
	24	74/F/ Left/37 y	FEV_1_, 1.47 L (75%); FVC, 1.83 L (70%); FEV_1_/FVC, 0.81 (106%)	CABG: 4 SVGs → LADA, lateral circumflex, high lateral circumflex, diagonal branch (left thoracotomy, on-pump)	None / Discharged on day 6; experienced stroke on day 18; died on day 22
Zhao et al [19]/2008	25	57/M/ Left /7 y	FEV_1_, 2.24 L (62%); FVC, 3.21 L (70%)	CABG: 2 SVGs → LADA, OMB, RCA (left thoracotomy, off-pump)	None / Discharged on day 9
Ghotkar et al [20]/2008	26	71/M/ Left /18 y	FEV_1_, 1.1 L (42%); FVC, 1.8 L (53%); FEV_1_/FVC, 0.79	CABG: SVG → LADA, PDA (median sternotomy, on-pump)	Excessive bleeding requiring re-exploration; atelectasis / Discharged on day 17
	27	77/F/ Right /13 mo	FEV_1_, 0.7 L (64%); FVC, 0.9 L (63%); FEV_1_/FVC, 1.04	Aortic valve replacement (median sternotomy, on-pump)	None / Not specified
Stamou et al [21]/2010	28	83/M/ Left /8 y	FEV_1_, (48%); **D**LCO, (77%)	Aortic valve replacement, CABG: SVGs (left thoracotomy, on-pump)	None / Discharged on day 5
Ushijima et al [22]/2011	29	82/M/ Left /20 y	FEV_1_, 1.28 L (64%); FVC, 1.89 L (64%); PaO_2_, 85 mmHg; PaCO_2_, 43 mmHg; pH, 7.40	CABG: LIMA, RA (Y) → LADA, posterolateral artery (left thoracotomy, off-pump)	None / Not specified

* **D**LCO = diffusing capacity of the lung for carbon monoxide; F = female; FEV_1_ = forced expiratory volume in one second; FVC = forced vital capacity; K_CO_ = transfer factor; LADA = left anterior descending artery; LCX = left anterior circumflex artery; LIMA = left internal mammary artery; M = male; OMB = obtuse marginal branch; 1OMB = first obtuse marginal branch; PaO_2_ = arterial oxygen partial pressure; PaCO_2_ = arterial carbon monoxide partial pressure; PAP = pulmonary arterial pressure; PDA = posterior descending artery; RA = radial artery; RCA = right coronary artery; RVSP = right ventricular systolic pressure; SVG = saphenous vein graft; VA = alveolar volume; (Y) = y-graft. Percentiles in parentheses refer to percentiles of predicted values. Days mentioned in the table refer to postoperative days.

All but one patient underwent one operation, apart from patient 22 who underwent two; therefore, 30 cardiac operations were performed on 29 patients. The most common operation performed was CABG alone (n = 21, 70%), followed by valvular surgery alone (n = 7, 23%) and combined CABG and valvular surgery (n = 2, 7%). Median sternotomy was the approach of choice in 80% of the cases (n = 24). Left thoracotomy was used in the rest 20% (n = 6), which involved only patients with previous left specifically pneumonectomy. Six (20%) underwent Off-pump CABG (OPCAG). In 3 of these operations left thoracotomy was the preferred approach.

FEV1 percentiles of predicted values were reported preoperatively in 23 cases and the mean percentile was 49% of predicted, ranging from 21% to 77% of predicted. In addition, preoperative FVC percentiles of predicted values were provided in 19 cases and the mean percentile was 51% of predicted, ranging from 27% to 70% of predicted. Preoperative arterial blood oxygen levels were provided in 12 cases and mean value was 74 mmHg, ranging from 60 to 92 mmHg. For arterial blood carbon dioxide, whose preoperative levels were reported in 12 cases, mean levels were 42 mmHg, ranging from 36 to 48 mmHg. Of the 30 operations performed, 11 (37%) were followed by complications, the most common being respiratory failure (n = 5, 17%), atrial fibrillation (n = 4, 13% of operations) and pneumothorax (n = 3, 10%), while 30-day mortality rate was 13% (n = 4).

## Discussion

The current review covers the database up to date regarding coronary revascularization and valvular procedures via open heart surgery in previously pneumonectomised patients. During our search we identified three articles [2,10,18] presenting a review of the subject. The first one by Medalion et al. [2] in 1994 was based on a worldwide survey of 118 members of the Society of Thoracic Surgeons, along with available data from the literature and one newly described patient. They presented collective data for 27 previously pneumonectomized patients who had undergone CABG and/or valve surgery. CABG was performed in 81% of cases, followed by mitral valve replacement in 19%. There were reported difficulties in exposing the circumflex marginal branches of 2 patients with left pneumonectomy. The most common postoperative complication was pneumothorax in 11% of patients, followed and postpericardiotomy syndrome in 4%, while the mortality rate was 7%.

In the second article by Diab et al [10] in 2001, the authors searched the MEDLINE database to identify six previously pneumonectomized patients from the literature and added one newly described patient to their review. All seven patients had undergone CABG. Forty-three percent of patients developed postoperative complications, the most common of them being pneumothorax and respiratory failure requiring re-intubation (29%) and prolonged ventilation. Overall mortality was 14%.

The third and most recent review was published by Stoller et al [18] in 2007. By searching the MEDLINE database from 1966 to 2006, they identified 15 patients with previous pneumonectomy who had undergone CABG and/or valve surgery, and also included 4 such patients from their institution’s Cardiovascular Information Registry. A total of 20 operations were performed in the 19 reviewed patients. CABG accounted for 75%, valve replacement or repair was performed in 20% and concomitant CABG and valve replacement/repair were performed in 5% of operations. Postoperative complications were reported in 50%, with the most common being respiratory failure and pneumothorax, in 25% and 10% of operations respectively. The 30-day mortality rate was 16%. Our current review includes the 15 patients reviewed by Stoller et al [18] (patients 1–5, 7–15 and 17), as well as the 4 newly described patients by Stoller et al [18] (patients 21–24). We extend our review by adding 10 more patients from the available up to date literature (patients 6, 16, 18–20 and 25–29) and believe, therefore, that all reported cases have been included and evaluated in this review.

It is well known that previously pneumonectomised patients have substantially reduced pulmonary capacities [23]. Although there is not precise data, most pneumonectomies had been performed for malignancy. In the current series, mean preoperative FEV1 and FVC values were 49% and 51% of predicted values respectively. Thus, preoperative respiratory physiotherapy, as well as steroids, bronchodilators and antibiotics should be used to improve postoperative morbidity and mortality rates in this high-risk group [24,25] We also believe that proper re-staging of the disease, in those pneumonectomised for lung carcinoma, should be undertaken preferably using a Positron Emission Tomography scan.

Pneumonectomy also incurs marked anatomical changes in the thorax [26,27]. This may necessitate deviations from standard protocols regarding open-heart surgery. For instance, in patients with previous left pneumonectomy, the heart and great vessels shift into the left chest and rotate [27]. This means that exposure of key sites for cardiopulmonary bypass cannulation, revascularization and valvular surgery may be easier via a left thoracotomy rather than a standard median sternotomy [7,18,19,21,22]. Stamou et al. [21] highlighted easy access to the aorta, the aortic root and the coronary arteries approaching the heart through a left thoracotomy in patients with previous left pneumonectomy. Nonetheless, since the degree of mediastinal shift varies greatly for each patient [26], alternative sites should be used if standard aortic cannulation is not possible and preoperative imaging tests, mainly chest CT scan, should be performed to assess best exposure of target sites. In our review, 6 patients with previous left pneumonectomy, underwent surgery via left thoracotomy, 5 underwent CABG alone (patients 8, 21, 24, 25 and 19) and 1 underwent combined CABG and aortic valve replacement (patient 28). Overall mortality rate for this subgroup of patients was 17%, n = 7.

Another consideration in patients with previous pneumonectomy is the ability to establish cardiopulmonary bypass (CPB). Berrizbeitia et al [4] reported difficulty during cannulation of inferior vena cava (IVC) in a patient with previous right pneumonectomy, because acute angulation had resulted from the rightward displacement of the right atrium, which could not be followed by the fixed IVC. Accordantly, axillary artery and femoral vein may also be considered as cannulation sites in patients where median sternotomy may be problematic [21].

It is worth noting that in our review, 6 patients (14, 15, 18, 21, 25 and 29) OPCAB, 3 of them via left thoracotomy (patients 21, 25 and 29). OPCAB, according to recent studies [28,29], has similar morbidity and mortality rates compared to standard CPB for CABG surgery, but boasts reduced incidence of systemic inflammatory response syndrome, blood clotting and blood loss, while claiming shorter ventilation and recovery periods. In previously pneumonectomised patients therefore, it may hold an advantage over CPB, since these patients already have impaired pulmonary function [30] and are at higher risk for CPB-related complications [10]. Ushijima et al [22] stated that OPCAB reduces postoperative pulmonary complications and eliminates the need for compromise between central venous system and target coronary site exposure.

There have been objections in the past literature concerning the use of the internal thoracic artery (ITA) to perform CABG in previously pneumonectomised patients [4,19,20]. For instance, the ITA may be difficult to harvest from the side of pneumonectomy and the pedicled graft may fail to reach its target site due to heart dislocation. In addition, the ITA may be subjected to excess stress and tension by the hyper-inflated lung. Moreover, harvesting of the ITA on the side of the intact lung may cause damage to the phrenic nerve10, while postoperative pain associated with ITA harvesting may deteriorate pulmonary function [31]. Nonetheless, Stoller et al [18] favoured its use, claiming favorable experience with the graft. Also, Bernet et al [15] suggested harvesting of a skeletonized ITA or performing a “LIMA fissure” technique [32] to alleviate stress on the ITA. Out of 21 CABG operations performed in our review, ITA graft was used in 8 (38%). Postoperative complications were reported in 2 of these cases and in 1 of them the outcome was fatal.

Prevalence of postoperative complications in the current series was 37% (n = 11), with respiratory failure being the most common (n = 5), requiring prolong ventilation and tracheostomy. This is justified by the reviewed patients’ compromised preoperative pulmonary reserves [23], along with the fact that anatomical changes of the chest may pose challenges on the surgical team, possibly leading to increased pump-related stress or even injury to chest structures [26]. Overall mortality rate in the current series was 13%, slightly less than the 16% reported in the last review by Stoller et al [18], but still high compared to patients with no such a severe comorbidity undergoing CABG or valve surgery [33,34]. Furthermore, major adverse cardiac and cardiovascular effects were not observed in a significantly higher rate among this cohort of patients. Unfortunately, there is no data to detect the long term prognosis, since most of the cases were focused on early postoperative outcome.

## Conclusion

In conclusion, the current series extends the previous review by Stoller et al [18] , summarizing the available data on 29 previously pneumonectomised patients who underwent 30 CABG and/or valvular operations. The gathered experience suggests that a carefully planned surgical strategy that incorporates modern techniques to alleviate patient stress, combined with specific standards regarding preoperative and postoperative management, contribute to successfully perform open heart revascularization and/or valvular procedures with acceptable but still high morbidity and mortality rates in previously pneumonectomised patients. Finally, in view of the new era of transfemoral aortic valve insertion (TAVI), this review it is likely to stimulate the existing argument as to whether, in these patients, a TAVI procedure should be preferred to an open aortic valve replacement.

## Competing interests

The author(s) declare that they have no competing interests.

## Authors’ contributions

AF collected the data and wrote the manuscript. AF helped in collecting the data. DD revised the manuscript. All authors read and approved the final manuscript.
